# Complete genome sequences of *Lichenicola* spp. GTC18330 and 18331 isolated from bee products in Gifu, Japan

**DOI:** 10.1128/mra.01442-25

**Published:** 2026-04-20

**Authors:** Masahiro Hayashi, Shin-ichiro Yokoyama, Takahiro Kashima, Tomomi Morimoto, Taketoshi Hata, Hiroe Maruyama, Hiroyuki Kono, Jun Yonetamari, Yoshinori Muto, Kaori Tanaka

**Affiliations:** 1 Institute for Glyco-core Research iGCORE, Gifu University 12785, Gifu City, Gifu, Japan; 2 Division of Anaerobe Research, Life Science Research Center, Gifu University 12785, Gifu City, Gifu, Japan; 3 Gifu University Center for Conservation of Microbial Genetic Resource, Gifu City, Gifu, Japan; 4 Gifu Prefectural Research Institute for Food Sciences, Gifu City, Gifu, Japan; 5 Nagaragawa R&D Center, API Co., Ltd. 371716 , Gifu City, Gifu, Japan; 6 Division of Clinical Laboratory, Gifu University Hospital 476117, Gifu City, Gifu, Japan; 7 United Graduate School of Drug Discovery and Medical Information Sciences, Gifu University 12785, Gifu City, Japan; Indiana University, Bloomington, Bloomington, Indiana, USA

**Keywords:** *Lichenicola*, bee products

## Abstract

We report the complete genome sequences of the *Lichenicola* strains GTC18330 and 18331 isolated from bee products in Gifu, Japan. The genomes comprise circular chromosomes measuring 3,633,834 bp (GTC18330) and 3,633,837 bp (GTC18331).

## ANNOUNCEMENT

*Lichenicola* species are members of the family *Acetobacteraceae* and are commonly isolated from the environment (1–3). In this study, strains GTC18330 and 18331 were isolated from bee bread (fermented pollen) and honey in Japan, as described by Moonmangmee et al. with modifications (4). Briefly, 1 g of each sample was inoculated into 50 mL of enrichment medium containing 0.5% d-sorbitol, 0.5% d-mannitol, 0.1% yeast extract, 0.1% polypeptone, 0.03% cycloheximide, and 1 M gluconate (pH 4.0) and incubated at 30°C for 7 days. The culture was spread on agar plates containing the same medium supplemented with 1.5% agar and 3 mg/100 mL bromocresol purple. After incubation at 30°C for 3 days, yellow colonies were selected for further experiments.

Genomic DNA was extracted from pelleted cells using the NucleoBond HMW DNA kit (MACHEREY-NAGEL, Germany). The genome was sequenced using PromethION 2i (Oxford Nanopore Technologies [ONT], Oxford, UK) for long-read and DNBSEQ (MGI Tech Co., Ltd., Shenzhen, China) for short-read sequencing (5–7). The same DNA extract was used for both platforms. A library was constructed with a Native Barcoding Kit (SQK-NBD114-24; ONT) without shearing for long-read sequencing. Small DNA fragments were removed using the Short Read Eliminator XS kit (Pacific Biosciences of California, Inc., Menlo Park, CA, USA). Sequencing was performed on PromethION 2i (ONT) with a FLO-PRO114M flow cell. Base calling was performed with Dorado v.7.2.14 (ultra-high-accuracy mode), and raw reads were trimmed and quality-filtered using NanoFilt v.2.7.1 (8) with parameters “-l 1000 -q 10 --headcrop 50.” For short reads, the library was constructed using the MGIEasy FS DNA Library Prep Set (MGI Tech), and 2 × 150 bp paired-end sequencing was performed on the DNBSEQ-G400 platform (MGI Tech). Reads were processed with fastp v0.20.1 (9) using the parameters “-q 30 -n 20 -t 1 -T 1.” Short-read quality was evaluated using fastp (9) and long-read quality was assessed using NanoPlot v1.32.1 (9). Short reads (with over 95.4% of bases >*Q*_30_) and long reads (mean read quality of 13.4) were assembled using Unicycler v.0.4.8 (10) for GTC18331. For GTC18330, long reads (mean read quality of 12.1) and short reads (with over 95.7% of bases >*Q*_30_) were assembled using Flye v.2.9 (11). Both assemblers were used with default settings. Assembly circularization and rotation were performed automatically in Unicycler and Flye. The contig graph was visualized with Bandage v0.8.1 (12), and the taxonomic integrity was confirmed using Blobtools v1.0 with short reads (13). Genome annotation was performed using the DDBJ Fast Annotation and Submission Tool (DFAST) (14). The assembly was rotated to start with the *dnaA* gene on the forward strand.

Table 1 summarizes the genomic information. Quality and genome statistics were computed using CheckM (v1.2.2) (15) and seqkit (v2.9.0) (16). CheckM confirmed that both genomes were 100% complete with 0.0% contamination.

**TABLE 1:** Information on the complete genome sequences of *Lichenicola* spp. strains isolated from bee products in Gifu, Japan

Parameter		Strain name	
		*Lichenicola* sp. GTC18330	*Lichenicola* sp. GTC18331
DNBSEQ sequencing^*a*^			
No. of reads		6,54,368	10,515,948
Size (kb)		1,013,155	1,577,392
Average coverage (×)		278.8	434.1
DRA accession no.		DRR791594	DRR791596
ONT sequencing^*a*^			
No. of reads		233,991	280,071
Size (kb)		695,787	905,027
Average read length (bp)		2,974	3,231
Average coverage (×)		191.5	249.1
*N*_50_		5,793.0	5,377.0
DRA accession no.		DRR791595	DRR791597
Assembly			
Assembly *N*_50_ (bp)^*c*^		3,633,834	3,633,837
Estimated genome completeness (%)^*d*^		100.0	100.0
Estimated genome contamination (%)^*d*^		0.0	0.0
Genome structure		One chromosome	One chromosome
DDBJ/GenBank accession no.		AP044853	AP045015
Genome size (bp)		3,633,834	3,633,837
GC content (%)		67.0	67.0
No. of coding sequences^*b*^		3,281	3,279
Number of rRNAs^*b*^		9	9
Number of tRNAs^*b*^		55	55
Number of CRISPRs^*b*^		0	0

^*a*^ DRA, DDBJ Sequence Read Archive.

^*b*^ DFAST, DDBJ Fast Annotation and Submission Tool.

^*c*^ Determined with seqkit v2.3.0.

^*d*^ Determined with CheckM v1.2.2.

## Data Availability

The genome sequences have been deposited in DDBJ under accession numbers AP044853 (GTC18330) and AP045015 (GTC18331). Raw reads are available in the DDBJ/Sequence Read Archive under accession numbers DRR791594 and DRR791595 (GTC18330), and DRR791596 and DRR791597 (GTC18331).
